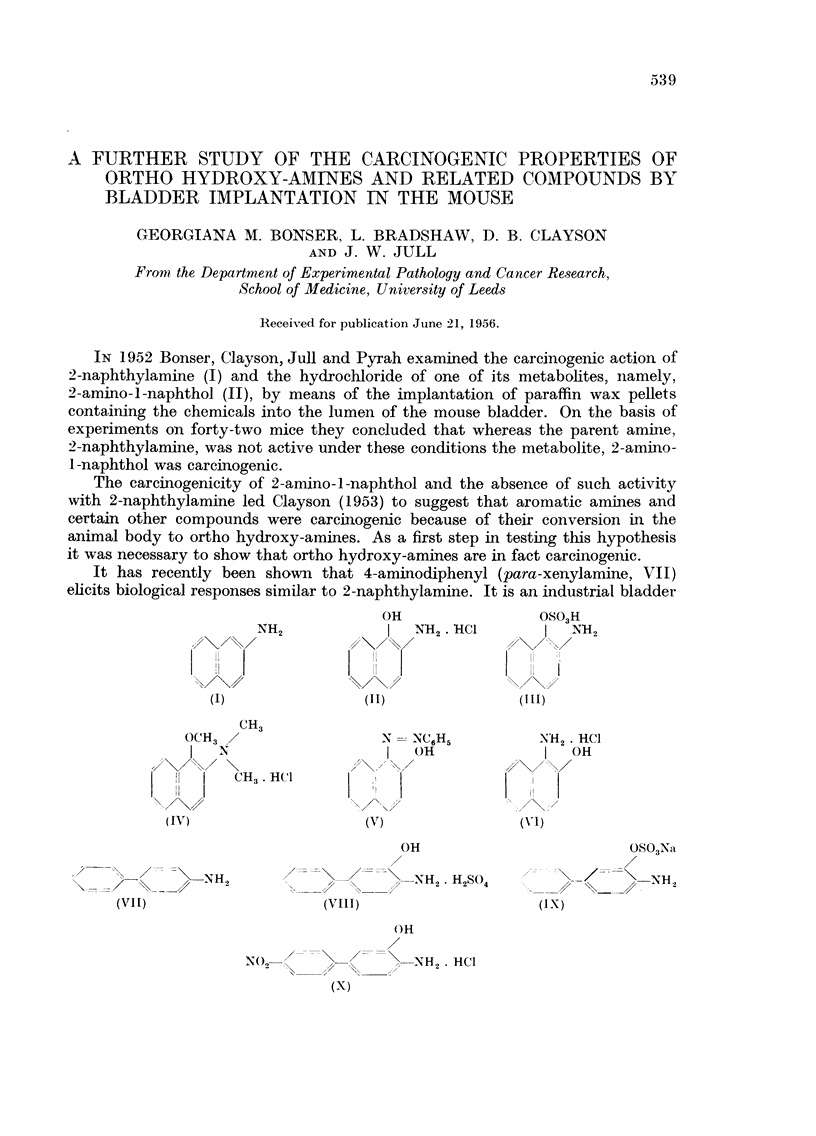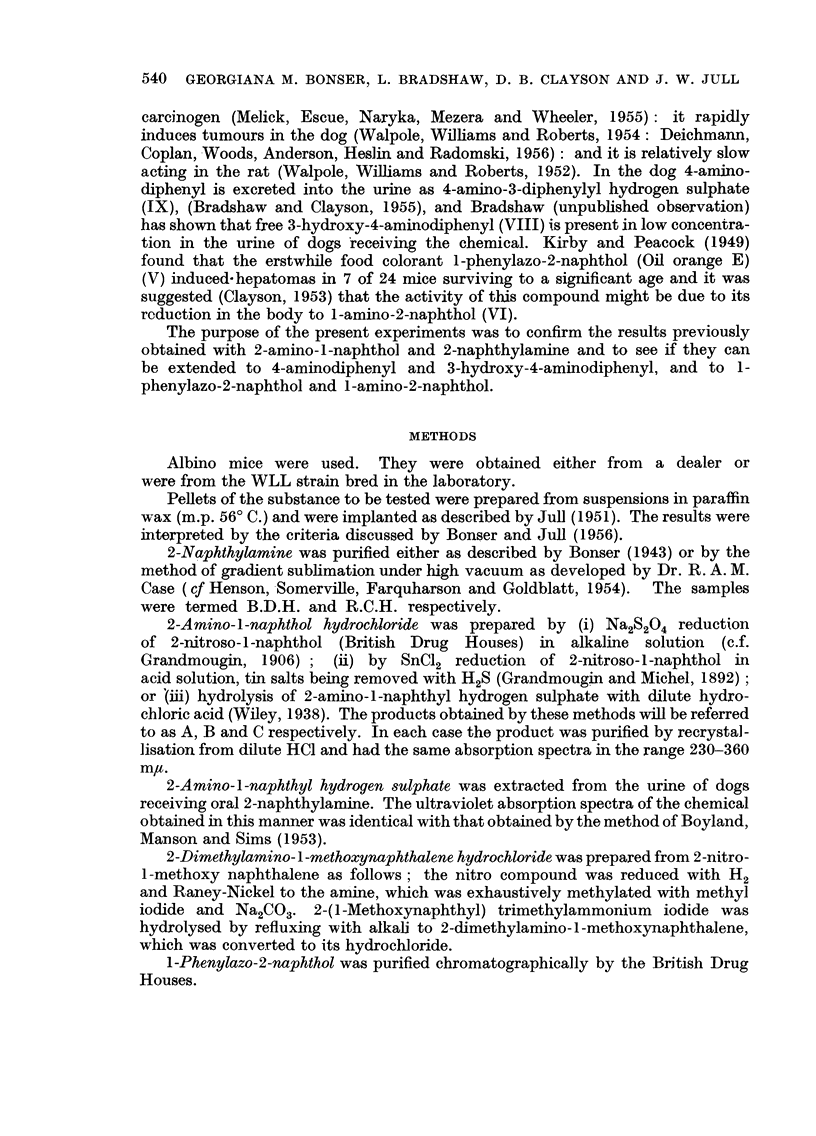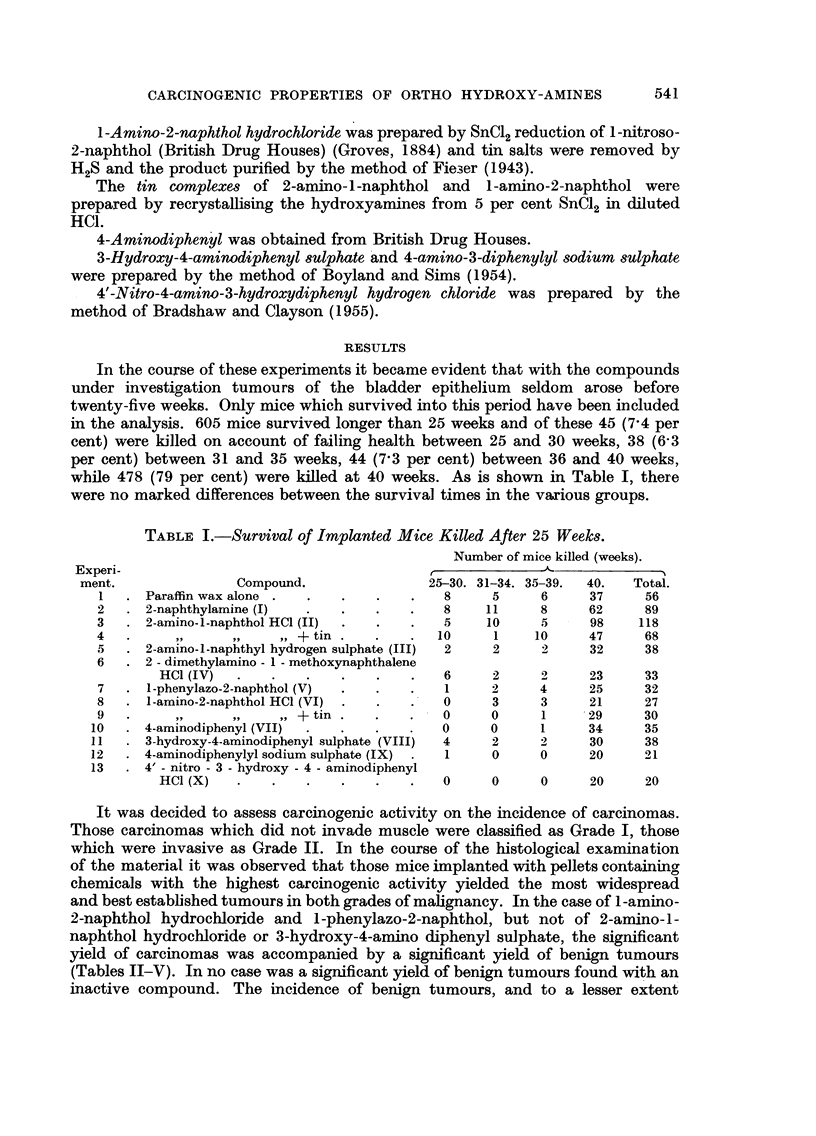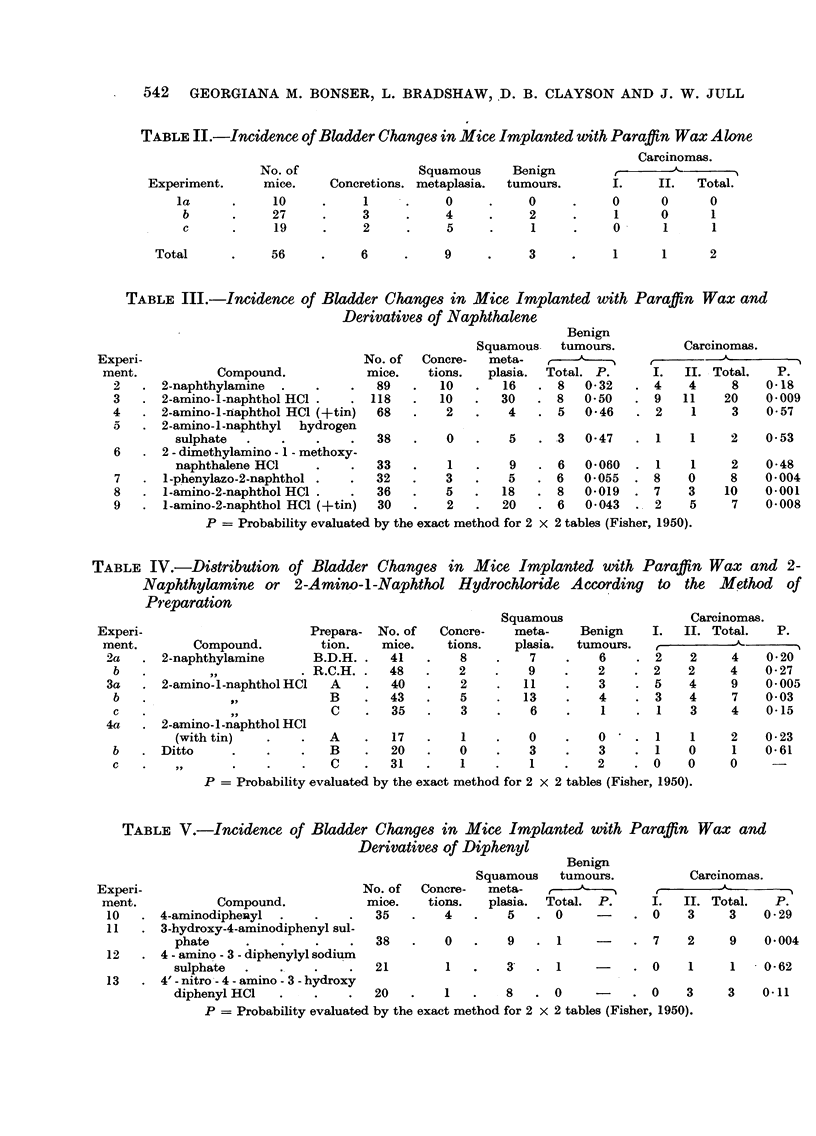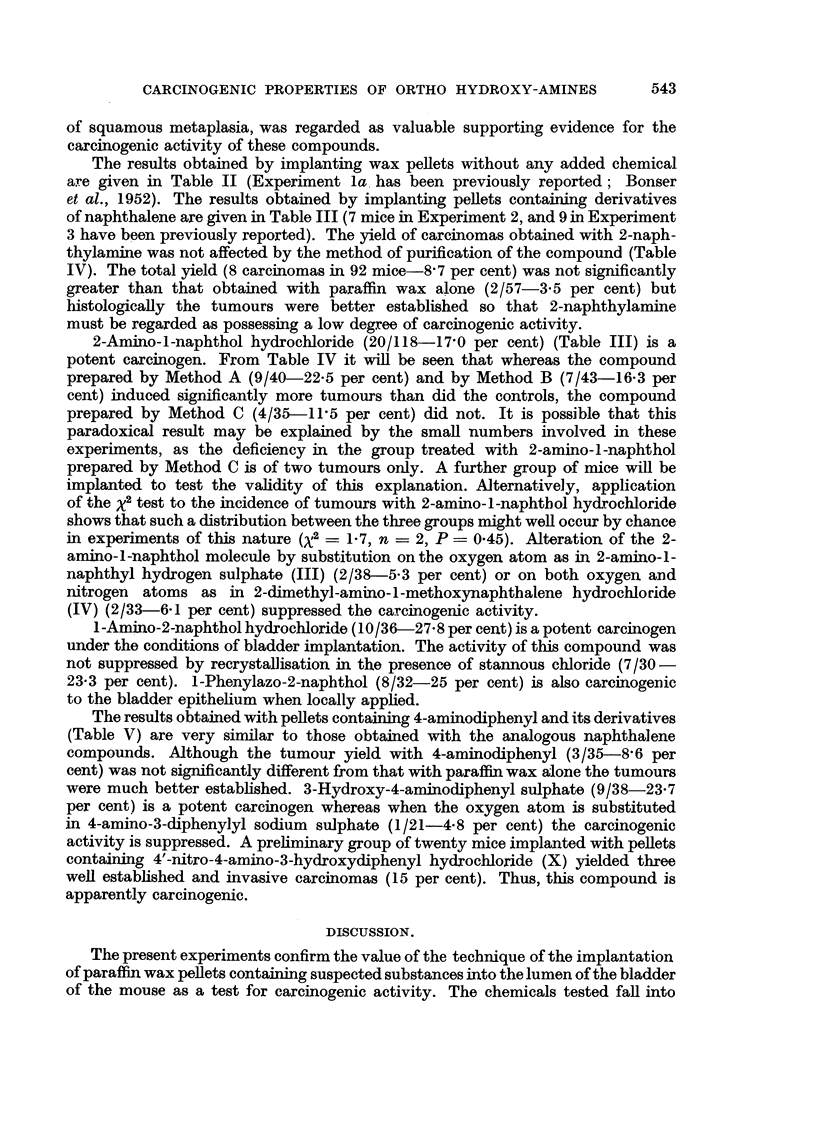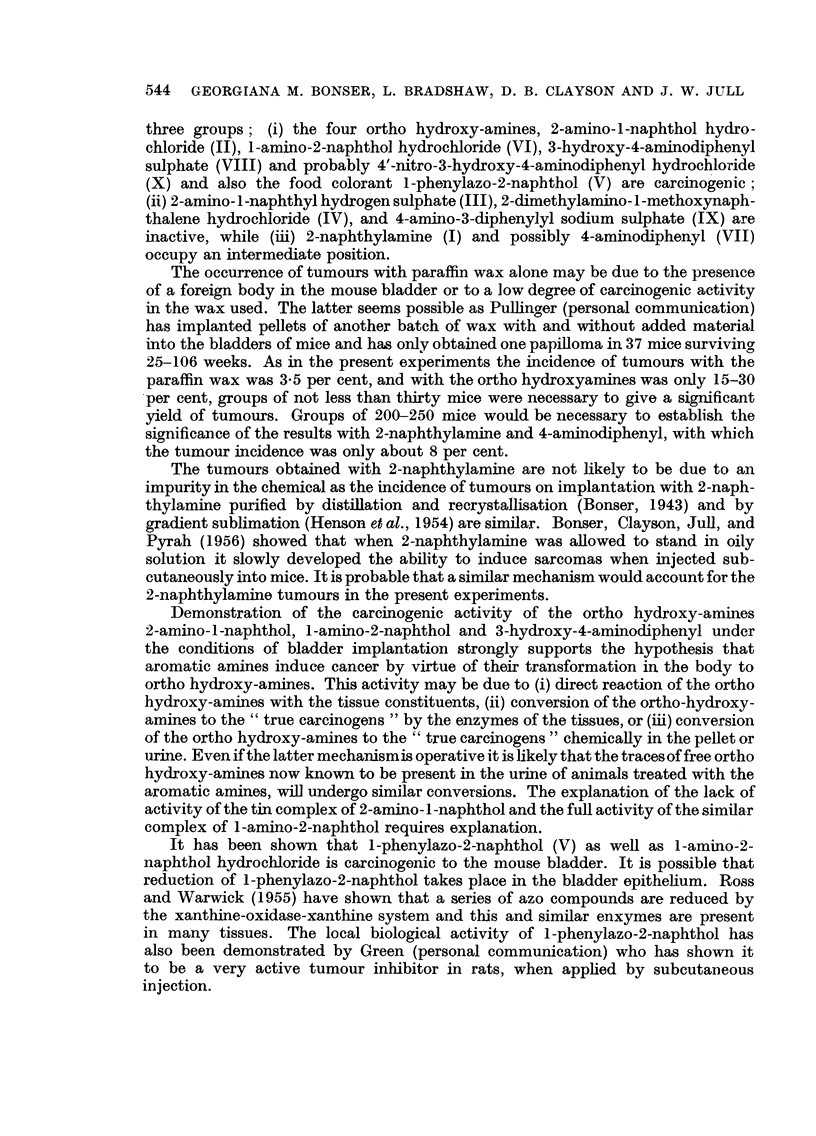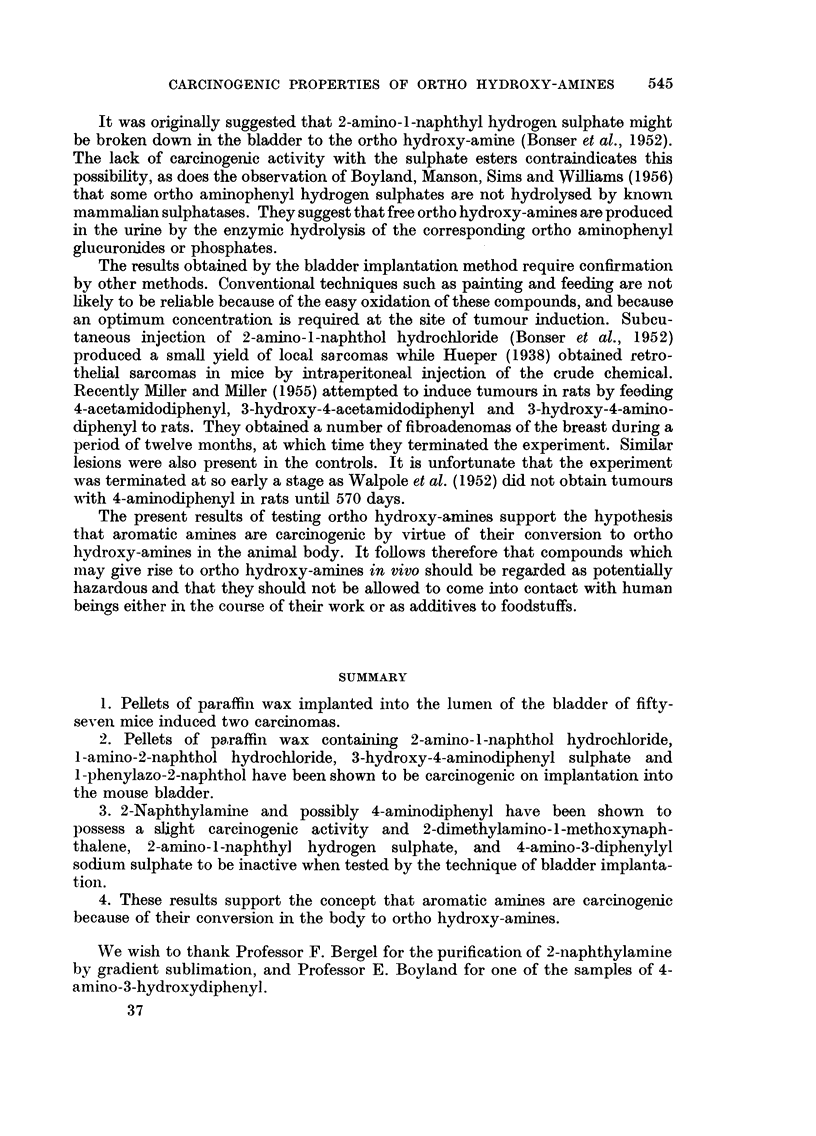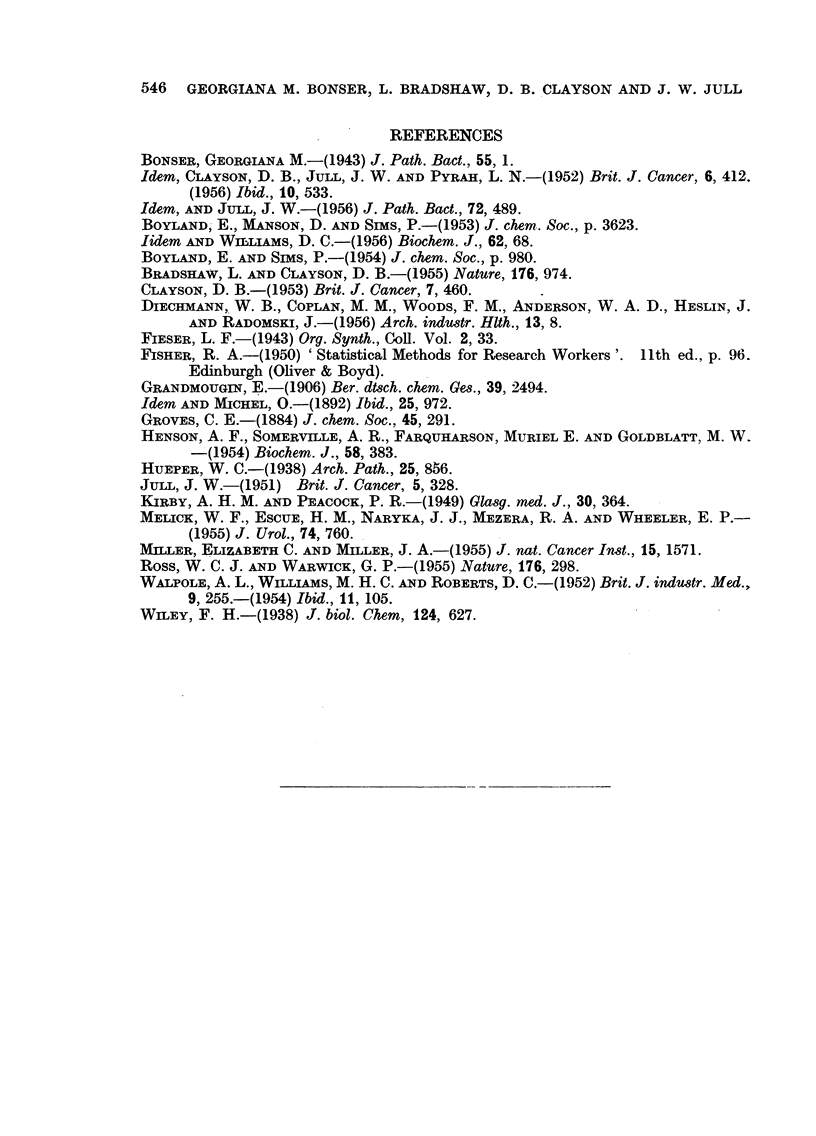# A Further Study of the Carcinogenic Properties of Ortho Hydroxy-Amines and Related Compounds by Bladder Implantation in the Mouse

**DOI:** 10.1038/bjc.1956.63

**Published:** 1956-09

**Authors:** Georgiana M. Bonser, L. Bradshaw, D. B. Clayson, J. W. Jull


### A FURTHER STUDY OF THE CARCINOGENIC PROPERTIES OF ORTHO HYDROXY-AMINES AND RELATED COMPOUNDS BY BLADDER IMPLANTATION IN THE MOUSE

GEORGIANA M. BONSER, L. BRADSHAW, D. B. CLAYSON AND J. W. JULL

*From the Department of Experimental Pathology and Cancer Research, School of Medicine, University of Leeds*

Received for publication June 21, 1956.

IN 1952 Bonser, Clayson, Jull and Pyrah examined the carcinogenic action of 2-naphthylamine (I) and the hydrochloride of one of its metabolites, namely, 2-amino-1-naphthol (II), by means of the implantation of paraffin wax pellets containing the chemicals into the lumen of the mouse bladder. On the basis of experiments on forty-two mice they concluded that whereas the parent amine, 2-naphthylamine, was not active under these conditions the metabolite, 2-amino-1-naphthol was carcinogenic.

The carcinogenicity of 2-amino-1-naphthol and the absence of such activity with 2-naphthylamine led Clayson (1953) to suggest that aromatic amines and certain other compounds were carcinogenic because of their conversion in the animal body to ortho hydroxy-amines. As a first step in testing this hypothesis it was necessary to show that ortho hydroxy-amines are in fact carcinogenic.

It has recently been shown that 4-aminodiphenyl (*para*-xenylamine, VII) elicits biological responses similar to 2-naphthylamine. It is an industrial bladder

(I)

(II)

(III)

(IV)

(V)

(VI)

(VII)

(VIII)

(IX)

(X)

carcinogen (Melick, Escue, Naryka, Mezera and Wheeler, 1955) : it rapidly induces tumours in the dog (Walpole, Williams and Roberts, 1954 : Deichmann, Coplan, Woods, Anderson, Heslin and Radomski, 1956) : and it is relatively slow acting in the rat (Walpole, Williams and Roberts, 1952). In the dog 4-aminodiphenyl is excreted into the urine as 4-amino-3-diphenylyl hydrogen sulphate (IX), (Bradshaw and Clayson, 1955), and Bradshaw (unpublished observation) has shown that free 3-hydroxy-4-aminodiphenyl (VIII) is present in low concentration in the urine of dogs receiving the chemical. Kirby and Peacock (1949) found that the erstwhile food colorant 1-phenylazo-2-naphthol (Oil orange E) (V) induced·hepatomas in 7 of 24 mice surviving to a significant age and it was suggested (Clayson, 1953) that the activity of this compound might be due to its reduction in the body to 1-amino-2-naphthol (VI).

The purpose of the present experiments was to confirm the results previously obtained with 2-amino-1-naphthol and 2-naphthylamine and to see if they can be extended to 4-aminodiphenyl and 3-hydroxy-4-aminodiphenyl, and to 1-phenylazo-2-naphthol and 1-amino-2-naphthol.

### METHODS

Albino mice were used. They were obtained either from a dealer or were from the WLL strain bred in the laboratory.

Pellets of the substance to be tested were prepared from suspensions in paraffin wax (m.p. 56° C.) and were implanted as described by Jull (1951). The results were interpreted by the criteria discussed by Bonser and Jull (1956).

2-*Naphthylamine* was purified either as described by Bonser (1943) or by the method of gradient sublimation under high vacuum as developed by Dr. R. A. M. Case (*cf* Henson, Somerville, Farquharson and Goldblatt, 1954). The samples were termed B.D.H. and R.C.H. respectively.

2-*Amino*-1-*naphthol hydrochloride* was prepared by (i) $Na_2S_2O_4$ reduction of 2-nitroso-1-naphthol (British Drug Houses) in alkaline solution (c.f. Grandmougin, 1906) ; (ii) by $SnCl_2$ reduction of 2-nitroso-1-naphthol in acid solution, tin salts being removed with $H_2S$ (Grandmougin and Michel, 1892) ; or (iii) hydrolysis of 2-amino-1-naphthyl hydrogen sulphate with dilute hydrochloric acid (Wiley, 1938). The products obtained by these methods will be referred to as A, B and C respectively. In each case the product was purified by recrystallisation from dilute HCl and had the same absorption spectra in the range 230–360 m$\mu$.

2-*Amino*-1-*naphthyl hydrogen sulphate* was extracted from the urine of dogs receiving oral 2-naphthylamine. The ultraviolet absorption spectra of the chemical obtained in this manner was identical with that obtained by the method of Boyland, Manson and Sims (1953).

2-*Dimethylamino*-1-*methoxynaphthalene hydrochloride* was prepared from 2-nitro-1-methoxy naphthalene as follows ; the nitro compound was reduced with $H_2$ and Raney-Nickel to the amine, which was exhaustively methylated with methyl iodide and $Na_2CO_3$. 2-(1-Methoxynaphthyl) trimethylammonium iodide was hydrolysed by refluxing with alkali to 2-dimethylamino-1-methoxynaphthalene, which was converted to its hydrochloride.

1-*Phenylazo*-2-*naphthol* was purified chromatographically by the British Drug Houses.

1-*Amino*-2-*naphthol hydrochloride* was prepared by $SnCl_2$ reduction of 1-nitroso-2-naphthol (British Drug Houses) (Groves, 1884) and tin salts were removed by $H_2S$ and the product purified by the method of Fieser (1943).

The *tin complexes* of 2-amino-1-naphthol and 1-amino-2-naphthol were prepared by recrystallising the hydroxyamines from 5 per cent $SnCl_2$ in diluted HCl.

4-*Aminodiphenyl* was obtained from British Drug Houses.

3-*Hydroxy*-4-*aminodiphenyl sulphate* and 4-*amino*-3-*diphenylyl sodium sulphate* were prepared by the method of Boyland and Sims (1954).

4′-*Nitro*-4-*amino*-3-*hydroxydiphenyl hydrogen chloride* was prepared by the method of Bradshaw and Clayson (1955).

### RESULTS

In the course of these experiments it became evident that with the compounds under investigation tumours of the bladder epithelium seldom arose before twenty-five weeks. Only mice which survived into this period have been included in the analysis. 605 mice survived longer than 25 weeks and of these 45 (7·4 per cent) were killed on account of failing health between 25 and 30 weeks, 38 (6·3 per cent) between 31 and 35 weeks, 44 (7·3 per cent) between 36 and 40 weeks, while 478 (79 per cent) were killed at 40 weeks. As is shown in Table I, there were no marked differences between the survival times in the various groups.

TABLE I.—*Survival of Implanted Mice Killed After* 25 *Weeks.*

| Experiment. | Compound. | Number of mice killed (weeks). 25–30. | 31–34. | 35–39. | 40. | Total. |
|---|---|---|---|---|---|---|
| 1 | Paraffin wax alone | 8 | 5 | 6 | 37 | 56 |
| 2 | 2-naphthylamine (I) | 8 | 11 | 8 | 62 | 89 |
| 3 | 2-amino-1-naphthol HCl (II) | 5 | 10 | 5 | 98 | 118 |
| 4 | ,, ,, ,, + tin | 10 | 1 | 10 | 47 | 68 |
| 5 | 2-amino-1-naphthyl hydrogen sulphate (III) | 2 | 2 | 2 | 32 | 38 |
| 6 | 2 - dimethylamino - 1 - methoxynaphthalene HCl (IV) | 6 | 2 | 2 | 23 | 33 |
| 7 | 1-phenylazo-2-naphthol (V) | 1 | 2 | 4 | 25 | 32 |
| 8 | 1-amino-2-naphthol HCl (VI) | 0 | 3 | 3 | 21 | 27 |
| 9 | ,, ,, ,, + tin | 0 | 0 | 1 | 29 | 30 |
| 10 | 4-aminodiphenyl (VII) | 0 | 0 | 1 | 34 | 35 |
| 11 | 3-hydroxy-4-aminodiphenyl sulphate (VIII) | 4 | 2 | 2 | 30 | 38 |
| 12 | 4-aminodiphenylyl sodium sulphate (IX) | 1 | 0 | 0 | 20 | 21 |
| 13 | 4′ - nitro - 3 - hydroxy - 4 - aminodiphenyl HCl (X) | 0 | 0 | 0 | 20 | 20 |

It was decided to assess carcinogenic activity on the incidence of carcinomas. Those carcinomas which did not invade muscle were classified as Grade I, those which were invasive as Grade II. In the course of the histological examination of the material it was observed that those mice implanted with pellets containing chemicals with the highest carcinogenic activity yielded the most widespread and best established tumours in both grades of malignancy. In the case of 1-amino-2-naphthol hydrochloride and 1-phenylazo-2-naphthol, but not of 2-amino-1-naphthol hydrochloride or 3-hydroxy-4-amino diphenyl sulphate, the significant yield of carcinomas was accompanied by a significant yield of benign tumours (Tables II–V). In no case was a significant yield of benign tumours found with an inactive compound. The incidence of benign tumours, and to a lesser extent

TABLE II.—*Incidence of Bladder Changes in Mice Implanted with Paraffin Wax Alone*

| Experiment. | No. of mice. | Concretions. | Squamous metaplasia. | Benign tumours. | Carcinomas. I. | Carcinomas. II. | Carcinomas. Total. |
|---|---|---|---|---|---|---|---|
| 1*a* | 10 | 1 | 0 | 0 | 0 | 0 | 0 |
| *b* | 27 | 3 | 4 | 2 | 1 | 0 | 1 |
| *c* | 19 | 2 | 5 | 1 | 0 | 1 | 1 |
| Total | 56 | 6 | 9 | 3 | 1 | 1 | 2 |

TABLE III.—*Incidence of Bladder Changes in Mice Implanted with Paraffin Wax and Derivatives of Naphthalene*

| Experiment. | Compound. | No. of mice. | Concretions. | Squamous metaplasia. | Benign tumours. Total. | Benign tumours. *P*. | Carcinomas. I. | Carcinomas. II. | Carcinomas. Total. | Carcinomas. P. |
|---|---|---|---|---|---|---|---|---|---|---|
| 2 | 2-naphthylamine | 89 | 10 | 16 | 8 | 0·32 | 4 | 4 | 8 | 0·18 |
| 3 | 2-amino-1-naphthol HCl | 118 | 10 | 30 | 8 | 0·50 | 9 | 11 | 20 | 0·009 |
| 4 | 2-amino-1-naphthol HCl (+tin) | 68 | 2 | 4 | 5 | 0·46 | 2 | 1 | 3 | 0·57 |
| 5 | 2-amino-1-naphthyl hydrogen sulphate | 38 | 0 | 5 | 3 | 0·47 | 1 | 1 | 2 | 0·53 |
| 6 | 2-dimethylamino-1-methoxy-naphthalene HCl | 33 | 1 | 9 | 6 | 0·060 | 1 | 1 | 2 | 0·48 |
| 7 | 1-phenylazo-2-naphthol | 32 | 3 | 5 | 6 | 0·055 | 8 | 0 | 8 | 0·004 |
| 8 | 1-amino-2-naphthol HCl | 36 | 5 | 18 | 8 | 0·019 | 7 | 3 | 10 | 0·001 |
| 9 | 1-amino-2-naphthol HCl (+tin) | 30 | 2 | 20 | 6 | 0·043 | 2 | 5 | 7 | 0·008 |

*P* = Probability evaluated by the exact method for 2 × 2 tables (Fisher, 1950).

TABLE IV.—*Distribution of Bladder Changes in Mice Implanted with Paraffin Wax and 2-Naphthylamine or 2-Amino-1-Naphthol Hydrochloride According to the Method of Preparation*

| Experiment. | Compound. | Preparation. | No. of mice. | Concretions. | Squamous metaplasia. | Benign tumours. | Carcinomas. I. | Carcinomas. II. | Carcinomas. Total. | P. |
|---|---|---|---|---|---|---|---|---|---|---|
| 2*a* | 2-naphthylamine | B.D.H. | 41 | 8 | 7 | 6 | 2 | 2 | 4 | 0·20 |
| *b* | ,, | R.C.H. | 48 | 2 | 9 | 2 | 2 | 2 | 4 | 0·27 |
| 3*a* | 2-amino-1-naphthol HCl | A | 40 | 2 | 11 | 3 | 5 | 4 | 9 | 0·005 |
| *b* | ,, | B | 43 | 5 | 13 | 4 | 3 | 4 | 7 | 0·03 |
| *c* | ,, | C | 35 | 3 | 6 | 1 | 1 | 3 | 4 | 0·15 |
| 4*a* | 2-amino-1-naphthol HCl (with tin) | A | 17 | 1 | 0 | 0 | 1 | 1 | 2 | 0·23 |
| *b* | Ditto | B | 20 | 0 | 3 | 3 | 1 | 0 | 1 | 0·61 |
| *c* | ,, | C | 31 | 1 | 1 | 2 | 0 | 0 | 0 | — |

*P* = Probability evaluated by the exact method for 2 × 2 tables (Fisher, 1950).

TABLE V.—*Incidence of Bladder Changes in Mice Implanted with Paraffin Wax and Derivatives of Diphenyl*

| Experiment. | Compound. | No. of mice. | Concretions. | Squamous metaplasia. | Benign tumours. Total. | Benign tumours. *P*. | Carcinomas. I. | Carcinomas. II. | Carcinomas. Total. | Carcinomas. *P*. |
|---|---|---|---|---|---|---|---|---|---|---|
| 10 | 4-aminodiphenyl | 35 | 4 | 5 | 0 | — | 0 | 3 | 3 | 0·29 |
| 11 | 3-hydroxy-4-aminodiphenyl sulphate | 38 | 0 | 9 | 1 | — | 7 | 2 | 9 | 0·004 |
| 12 | 4-amino-3-diphenylyl sodium sulphate | 21 | 1 | 3 | 1 | — | 0 | 1 | 1 | 0·62 |
| 13 | 4′-nitro-4-amino-3-hydroxy diphenyl HCl | 20 | 1 | 8 | 0 | — | 0 | 3 | 3 | 0·11 |

*P* = Probability evaluated by the exact method for 2 × 2 tables (Fisher, 1950).

of squamous metaplasia, was regarded as valuable supporting evidence for the carcinogenic activity of these compounds.

The results obtained by implanting wax pellets without any added chemical are given in Table II (Experiment 1*a* has been previously reported; Bonser *et al.*, 1952). The results obtained by implanting pellets containing derivatives of naphthalene are given in Table III (7 mice in Experiment 2, and 9 in Experiment 3 have been previously reported). The yield of carcinomas obtained with 2-naphthylamine was not affected by the method of purification of the compound (Table IV). The total yield (8 carcinomas in 92 mice—8·7 per cent) was not significantly greater than that obtained with paraffin wax alone (2/57—3·5 per cent) but histologically the tumours were better established so that 2-naphthylamine must be regarded as possessing a low degree of carcinogenic activity.

2-Amino-1-naphthol hydrochloride (20/118—17·0 per cent) (Table III) is a potent carcinogen. From Table IV it will be seen that whereas the compound prepared by Method A (9/40—22·5 per cent) and by Method B (7/43—16·3 per cent) induced significantly more tumours than did the controls, the compound prepared by Method C (4/35—11·5 per cent) did not. It is possible that this paradoxical result may be explained by the small numbers involved in these experiments, as the deficiency in the group treated with 2-amino-1-naphthol prepared by Method C is of two tumours only. A further group of mice will be implanted to test the validity of this explanation. Alternatively, application of the $\chi^2$ test to the incidence of tumours with 2-amino-1-naphthol hydrochloride shows that such a distribution between the three groups might well occur by chance in experiments of this nature ($\chi^2 = 1{\cdot}7$, $n = 2$, $P = 0{\cdot}45$). Alteration of the 2-amino-1-naphthol molecule by substitution on the oxygen atom as in 2-amino-1-naphthyl hydrogen sulphate (III) (2/38—5·3 per cent) or on both oxygen and nitrogen atoms as in 2-dimethyl-amino-1-methoxynaphthalene hydrochloride (IV) (2/33—6·1 per cent) suppressed the carcinogenic activity.

1-Amino-2-naphthol hydrochloride (10/36—27·8 per cent) is a potent carcinogen under the conditions of bladder implantation. The activity of this compound was not suppressed by recrystallisation in the presence of stannous chloride (7/30—23·3 per cent). 1-Phenylazo-2-naphthol (8/32—25 per cent) is also carcinogenic to the bladder epithelium when locally applied.

The results obtained with pellets containing 4-aminodiphenyl and its derivatives (Table V) are very similar to those obtained with the analogous naphthalene compounds. Although the tumour yield with 4-aminodiphenyl (3/35—8·6 per cent) was not significantly different from that with paraffin wax alone the tumours were much better established. 3-Hydroxy-4-aminodiphenyl sulphate (9/38—23·7 per cent) is a potent carcinogen whereas when the oxygen atom is substituted in 4-amino-3-diphenylyl sodium sulphate (1/21—4·8 per cent) the carcinogenic activity is suppressed. A preliminary group of twenty mice implanted with pellets containing 4′-nitro-4-amino-3-hydroxydiphenyl hydrochloride (X) yielded three well established and invasive carcinomas (15 per cent). Thus, this compound is apparently carcinogenic.

### DISCUSSION.

The present experiments confirm the value of the technique of the implantation of paraffin wax pellets containing suspected substances into the lumen of the bladder of the mouse as a test for carcinogenic activity. The chemicals tested fall into three groups ; (i) the four ortho hydroxy-amines, 2-amino-1-naphthol hydrochloride (II), 1-amino-2-naphthol hydrochloride (VI), 3-hydroxy-4-aminodiphenyl sulphate (VIII) and probably 4'-nitro-3-hydroxy-4-aminodiphenyl hydrochloride (X) and also the food colorant 1-phenylazo-2-naphthol (V) are carcinogenic ; (ii) 2-amino-1-naphthyl hydrogen sulphate (III), 2-dimethylamino-1-methoxynaphthalene hydrochloride (IV), and 4-amino-3-diphenylyl sodium sulphate (IX) are inactive, while (iii) 2-naphthylamine (I) and possibly 4-aminodiphenyl (VII) occupy an intermediate position.

The occurrence of tumours with paraffin wax alone may be due to the presence of a foreign body in the mouse bladder or to a low degree of carcinogenic activity in the wax used. The latter seems possible as Pullinger (personal communication) has implanted pellets of another batch of wax with and without added material into the bladders of mice and has only obtained one papilloma in 37 mice surviving 25–106 weeks. As in the present experiments the incidence of tumours with the paraffin wax was 3·5 per cent, and with the ortho hydroxyamines was only 15–30 per cent, groups of not less than thirty mice were necessary to give a significant yield of tumours. Groups of 200–250 mice would be necessary to establish the significance of the results with 2-naphthylamine and 4-aminodiphenyl, with which the tumour incidence was only about 8 per cent.

The tumours obtained with 2-naphthylamine are not likely to be due to an impurity in the chemical as the incidence of tumours on implantation with 2-naphthylamine purified by distillation and recrystallisation (Bonser, 1943) and by gradient sublimation (Henson *et al.*, 1954) are similar. Bonser, Clayson, Jull, and Pyrah (1956) showed that when 2-naphthylamine was allowed to stand in oily solution it slowly developed the ability to induce sarcomas when injected subcutaneously into mice. It is probable that a similar mechanism would account for the 2-naphthylamine tumours in the present experiments.

Demonstration of the carcinogenic activity of the ortho hydroxy-amines 2-amino-1-naphthol, 1-amino-2-naphthol and 3-hydroxy-4-aminodiphenyl under the conditions of bladder implantation strongly supports the hypothesis that aromatic amines induce cancer by virtue of their transformation in the body to ortho hydroxy-amines. This activity may be due to (i) direct reaction of the ortho hydroxy-amines with the tissue constituents, (ii) conversion of the ortho-hydroxy-amines to the " true carcinogens " by the enzymes of the tissues, or (iii) conversion of the ortho hydroxy-amines to the " true carcinogens " chemically in the pellet or urine. Even if the latter mechanism is operative it is likely that the traces of free ortho hydroxy-amines now known to be present in the urine of animals treated with the aromatic amines, will undergo similar conversions. The explanation of the lack of activity of the tin complex of 2-amino-1-naphthol and the full activity of the similar complex of 1-amino-2-naphthol requires explanation.

It has been shown that 1-phenylazo-2-naphthol (V) as well as 1-amino-2-naphthol hydrochloride is carcinogenic to the mouse bladder. It is possible that reduction of 1-phenylazo-2-naphthol takes place in the bladder epithelium. Ross and Warwick (1955) have shown that a series of azo compounds are reduced by the xanthine-oxidase-xanthine system and this and similar enxymes are present in many tissues. The local biological activity of 1-phenylazo-2-naphthol has also been demonstrated by Green (personal communication) who has shown it to be a very active tumour inhibitor in rats, when applied by subcutaneous injection.

It was originally suggested that 2-amino-1-naphthyl hydrogen sulphate might be broken down in the bladder to the ortho hydroxy-amine (Bonser *et al.*, 1952). The lack of carcinogenic activity with the sulphate esters contraindicates this possibility, as does the observation of Boyland, Manson, Sims and Williams (1956) that some ortho aminophenyl hydrogen sulphates are not hydrolysed by known mammalian sulphatases. They suggest that free ortho hydroxy-amines are produced in the urine by the enzymic hydrolysis of the corresponding ortho aminophenyl glucuronides or phosphates.

The results obtained by the bladder implantation method require confirmation by other methods. Conventional techniques such as painting and feeding are not likely to be reliable because of the easy oxidation of these compounds, and because an optimum concentration is required at the site of tumour induction. Subcutaneous injection of 2-amino-1-naphthol hydrochloride (Bonser *et al.*, 1952) produced a small yield of local sarcomas while Hueper (1938) obtained retrothelial sarcomas in mice by intraperitoneal injection of the crude chemical. Recently Miller and Miller (1955) attempted to induce tumours in rats by feeding 4-acetamidodiphenyl, 3-hydroxy-4-acetamidodiphenyl and 3-hydroxy-4-aminodiphenyl to rats. They obtained a number of fibroadenomas of the breast during a period of twelve months, at which time they terminated the experiment. Similar lesions were also present in the controls. It is unfortunate that the experiment was terminated at so early a stage as Walpole *et al.* (1952) did not obtain tumours with 4-aminodiphenyl in rats until 570 days.

The present results of testing ortho hydroxy-amines support the hypothesis that aromatic amines are carcinogenic by virtue of their conversion to ortho hydroxy-amines in the animal body. It follows therefore that compounds which may give rise to ortho hydroxy-amines *in vivo* should be regarded as potentially hazardous and that they should not be allowed to come into contact with human beings either in the course of their work or as additives to foodstuffs.

### SUMMARY

1. Pellets of paraffin wax implanted into the lumen of the bladder of fifty-seven mice induced two carcinomas.

2. Pellets of paraffin wax containing 2-amino-1-naphthol hydrochloride, 1-amino-2-naphthol hydrochloride, 3-hydroxy-4-aminodiphenyl sulphate and 1-phenylazo-2-naphthol have been shown to be carcinogenic on implantation into the mouse bladder.

3. 2-Naphthylamine and possibly 4-aminodiphenyl have been shown to possess a slight carcinogenic activity and 2-dimethylamino-1-methoxynaphthalene, 2-amino-1-naphthyl hydrogen sulphate, and 4-amino-3-diphenylyl sodium sulphate to be inactive when tested by the technique of bladder implantation.

4. These results support the concept that aromatic amines are carcinogenic because of their conversion in the body to ortho hydroxy-amines.

We wish to thank Professor F. Bergel for the purification of 2-naphthylamine by gradient sublimation, and Professor E. Boyland for one of the samples of 4-amino-3-hydroxydiphenyl.

### REFERENCES

BONSER, GEORGIANA M.—(1943) *J. Path. Bact.*, **55**, 1.

*Idem*, CLAYSON, D. B., JULL, J. W. AND PYRAH, L. N.—(1952) *Brit. J. Cancer*, **6**, 412. (1956) *Ibid.*, **10**, 533.

*Idem*, AND JULL, J. W.—(1956) *J. Path. Bact.*, **72**, 489.

BOYLAND, E., MANSON, D. AND SIMS, P.—(1953) *J. chem. Soc.*, p. 3623.

*Iidem* AND WILLIAMS, D. C.—(1956) *Biochem. J.*, **62**, 68.

BOYLAND, E. AND SIMS, P.—(1954) *J. chem. Soc.*, p. 980.

BRADSHAW, L. AND CLAYSON, D. B.—(1955) *Nature*, **176**, 974.

CLAYSON, D. B.—(1953) *Brit. J. Cancer*, **7**, 460.

DIECHMANN, W. B., COPLAN, M. M., WOODS, F. M., ANDERSON, W. A. D., HESLIN, J. AND RADOMSKI, J.—(1956) *Arch. industr. Hlth.*, **13**, 8.

FIESER, L. F.—(1943) *Org. Synth.*, Coll. Vol. **2**, 33.

FISHER, R. A.—(1950) 'Statistical Methods for Research Workers'. 11th ed., p. 96. Edinburgh (Oliver & Boyd).

GRANDMOUGIN, E.—(1906) *Ber. dtsch. chem. Ges.*, **39**, 2494.

*Idem* AND MICHEL, O.—(1892) *Ibid.*, **25**, 972.

GROVES, C. E.—(1884) *J. chem. Soc.*, **45**, 291.

HENSON, A. F., SOMERVILLE, A. R., FARQUHARSON, MURIEL E. AND GOLDBLATT, M. W. —(1954) *Biochem. J.*, **58**, 383.

HUEPER, W. C.—(1938) *Arch. Path.*, **25**, 856.

JULL, J. W.—(1951) *Brit. J. Cancer*, **5**, 328.

KIRBY, A. H. M. AND PEACOCK, P. R.—(1949) *Glasg. med. J.*, **30**, 364.

MELICK, W. F., ESCUE, H. M., NARYKA, J. J., MEZERA, R. A. AND WHEELER, E. P.—(1955) *J. Urol.*, **74**, 760.

MILLER, ELIZABETH C. AND MILLER, J. A.—(1955) *J. nat. Cancer Inst.*, **15**, 1571.

ROSS, W. C. J. AND WARWICK, G. P.—(1955) *Nature*, **176**, 298.

WALPOLE, A. L., WILLIAMS, M. H. C. AND ROBERTS, D. C.—(1952) *Brit. J. industr. Med.*, **9**, 255.—(1954) *Ibid.*, **11**, 105.

WILEY, F. H.—(1938) *J. biol. Chem*, **124**, 627.